# Chronic non-specific abdominal complaints in general practice: a prospective study on management, patient health status and course of complaints

**DOI:** 10.1186/1471-2296-7-12

**Published:** 2006-03-03

**Authors:** Henriëtte AM van den Heuvel-Janssen, Jeroen AJ Borghouts, Jean WM Muris, Bart W Koes, Lex M Bouter, J André Knottnerus

**Affiliations:** 1 Department of General Practice, Research Institute Caphri, Maastricht University, Maastricht, The Netherlands; 2 Academy of Healthcare, AVANS Uniersity of Professional Education, Breda, The Netherlands; 3 Department of General Practice, Erasmus University Medical Centre, Rotterdam, The Netherlands

## Abstract

**Background:**

While in general practice chronic non-specific abdominal complaints are common, there is insufficient data on the clinical course and the management of these complaints. Aim of this study was to present a primary care based profile of these chronic complaints including health care involvement, health status and clinical course.

**Methods:**

Thirty general practitioners (GPs) and patients from their practices participated in a prospective follow-up study. All patients and GPs were asked to complete questionnaires at baseline and at 6, 12 and 18 months of follow-up. The GPs provided information on diagnostic and therapeutic management and on referral concerning 619 patients with chronic non-specific abdominal complaints, while 291 patients provided information about health status and clinical course of the complaints.

**Results:**

When asked after 18 months of follow-up, 51,7% of the patients reported an equal or worsened severity of complaints. General health perception was impaired and patients had high scores on SCL-anxiety and SCL-depression scales. Diagnostic tests other than physical examination and laboratory tests were not frequently used. Medication was the most frequent type of treatment. The persistence of chronic non-specific abdominal complaints was quite stable.

**Conclusion:**

Once non-specific chronic abdominal complaints have become labelled as chronic by the attending physician, little improvement can be expected. The impact on patients' physiological and psychological well-being is large. GPs use a variety of diagnostic and therapeutic strategies. Research into the evidence base of currently applied management strategies is recommended.

## Background

Patients with non-specific abdominal complaints comprise a large proportion of primary care and gastroenterology practice [1-6]. Annually 15 out of every 1000 registered patients visit their general practitioner (GP) for new abdominal complaints [7]. Since chronic non-specific abdominal complaints are mainly managed by the GP, it is of importance to know how general practitioners deal with these complaints, especially since results, concerning management and prognosis, from more selected, e.g. hospital based, populations cannot be generalised to primary care [8]. Data on diagnostic and therapeutic strategies chosen in primary care are of importance for the development of tailor-made guidelines for the management of these complaints. However, despite this importance, little detailed data are available. Also, the clinical course of chronic non-specific abdominal complaints in general practice is not well documented. Reviews showed that no studies have been carried out on the clinical course of irritable bowel syndrome (IBS) in primary care [9] and that only 3 studies on the clinical course of non-ulcer dyspepsia (NUD) in primary care were performed [10].

In summary, the understanding of the management and the course of non-specific abdominal complaints in family practice seems to be rather poor. The aim of this study is to present a primary care based profile of chronic non-specific abdominal complaints in family practice including health care involvement, patients' perspective and clinical course.

The following research questions were formulated:

1. What is the health care involvement regarding patients with chronic non-specific abdominal complaints in primary care, including GP-visits, diagnostic and therapeutic management and referrals?

2. What is the course of the complaints and what are its determinants?

3. Do IBS, NUD and other abdominal complaints differ with respect to the former two questions?

## Methods

### • Patients and procedures

The GPs and patients who participated in this study were recruited from the Registration Network Family Practices (RNH) of the Maastricht University in the Netherlands [11]. The Registration Network provides a computerised anonymous database containing certain patient characteristics and relevant health problems. Health problems are defined as 'anything that has required, does or may require health care management and has affected or could significantly affect a persons physical or emotional well-being'. The RNH GPs only register permanent problems (no recovery expected), chronic problems (duration longer than six months) and/or recurrent problems (more than three recurrences within a six-month period). The health problems are coded using the International Classification of Primary Care (ICPC) [12] using diagnostic criteria based on the International Classification of Health Problems in Primary Care (ICHPPC-2) [13]. All information concerning each patient including data on diagnostic and therapeutic modalities are stored in electronic medical records in chronological sequence. The general practitioners continuously update the patient characteristics and problem lists of all their registered patients. The patient population is very similar to the Dutch general population regarding age, gender, type of health insurance and level of education [14].

From the RNH database potentially eligible patients were identified using five relevant ICPC codes:

1. Abdominal pain without organic explanation (ICPC codes D01, D02 and D06) and/or

2. Non-ulcer dyspepsia (ICPC code D87) and/or

3. Irritable bowel syndrome (ICPC code D93)

The purpose of this process was to select a cohort of patients with chronic non-specific abdominal complaints in general practice. The patients were registered with a total of 30 GPs working in 16 practices.

Patients were included on the basis of the following criteria: 1) abdominal pain without organic explanation and/or non-ulcer dyspepsia and/or irritable bowel syndrome (ICPC codes D01, D02, D06, D87 or D93), 2) at baseline, symptoms had to be present for at least six months, 3) there was no evidence of an organic cause of the symptoms and 4) patients had to be 18–70 years of age.

Patients were excluded when there was a malignancy of the gastrointestinal tract.

Eligible patients were invited by their GP to participate in the study. The GP and the first author checked in- and exclusion criteria.

All patients and GPs were asked to complete a questionnaire at baseline and after 6, 12 and 18 months. The baseline questionnaire covered the previous 12 months and each follow-up questionnaire covered the previous 6 months.

### • Instruments

The GP questionnaires covered variables such as age, gender, diagnosis, comorbidity, diagnostic modalities, therapeutic interventions and new referrals to specialists for the complaints at issue.

The patient questionnaires included variables such as duration, localisation, frequency and severity of the complaints. Global subjective improvement was categorised in: improvement versus no improvement and/or worsening as stated by the patient concerning the previous 6 month-period. The severity of the abdominal pain was measured on an 11-point ordinal scale ranging from 0 (no pain) to 10 (very severe pain).

The Nottingham Health Profile (NHP) was used to assess the general health perception. The first section of the NHP was used, consisting of 6 categories: energy, pain, emotional reactions, sleep, physical mobility and social isolation. Higher scores indicate more health problems. The original versions of the NHP and the Dutch translation have been demonstrated to be both reliable and valid. [15,16]

Subscales of the Symptom Checklist 90 (SCL-90) were used to assess depression and anxiety. Higher scores indicate more mental health problems. The original versions of the SCL-90 as well as the Dutch translation have both been shown to be valid and reliable [17,18].

The Gastrointestinal Symptom Rating Scale (GSRS) was used to assess the severity of gastrointestinal symptoms. The GSRS includes 15 items and uses a 7-graded Likert scale, where 1 presents the most positive option and 7 the most negative one. The higher the scores, the more pronounced are the symptoms. The items have been grouped in 5 dimensions: indigestion syndrome, diarrhoea syndrome, obstipation syndrome, abdominal pain syndrome and reflux syndrome. The original English version of the rating scale is interview-based. For the present study it was translated into Dutch and modified into a self-administered questionnaire. The original (English) version of the GSRS has been demonstrated to be reliable and valid [19,20].

### • Statistics

Descriptive statistics were used to present the course of complaints (pain intensity, global improvement, number of visits, NHP, SCL and GSRS), the frequencies of diagnostic and therapeutic interventions and the referrals over the 18-month follow-up.

Differences between subgroups were analysed by using the Chi-square test statistic (for categorical variables), the Student t test statistic (for continuous variables), ANOVA for three groups' comparisons, and the Mann-Whitney test statistic (for non-parametrical comparisons). A two-sided p-value of 0.05 was used as a criterion for statistical significance. Multivariable regression analysis was performed to detect possible prognostic determinants of the clinical course (SPSS 8.0).

• Ethical approval: for this study design ethical approval was not required.

## Results

### • Response

The study population initially consisted of 644 patients with chronic non-specific abdominal complaints. Data were collected in a period from 1997–1999. The GPs (n = 30) filled out and returned 628 questionnaires (97.5%). Of these 628 patients 9 did not meet our inclusion criteria because 8 were over 70 years of age and one was younger than 18 years of age. These patients were excluded from the analysis. Of these 619 patients 291 (47%) filled out and returned the baseline questionnaire. Thus, the final study population consisted of 619 patients with chronic non-specific abdominal complaints, with 291 subjects with data available from both patients and GP ("responders") and 328 subjects with data available from GP only ("non-responders").

The response rates for the GP questionnaires after 6, 12 and 18 months were 96.8%, 94.0% and 91.6%, respectively. In the follow-up period of 18 months 11 patients removed, 1 patient died, 30 patients were excluded from the study because an organic cause was found to explain the complaints and 10 patients were lost-to-follow-up without known reason.

The response rates for the patient questionnaires after 6, 12 and 18 months were 83.8%, 82.8% and 79.0%, respectively.

### • Patient characteristics

Mean age of the responders at baseline (n = 291) was 46.4 years (SD 12.8), 34.4% were men. We reported earlier that the non-responders were significantly more frequently male and significantly younger, had significantly longer duration of complaints, and paid significantly less visits to their GP [21].

### • Course of complaints

Table 1 shows the percentage of patients that report improvement in their chronic non-specific abdominal complaints, the mean GSRS scores, the mean scores on the SCL scales for anxiety and depression and the mean scores on the NHP scales, at baseline and after 18 months of follow-up. At baseline 55.2% of the patients did report improvement of their complaints during the previous 12 months. When asked after 18 months of follow-up this percentage went to 48,3%. Patients with IBS and other abdominal complaints reported less improvement than patients with NUD. Patients with IBS scored higher on the anxiety and depression scales than patients with NUD, and scored worse on NHP-sleep than patients with other abdominal complaints.

**Table 1:** Course of complaints for total study population and for Non Ulcer Dyspepsia (NUD), IBS and Other. (patient questionnaire data)

	Baseline				18 months				
	Total (n = 291)	NUD (n = 36)	IBS (n = 125)	Other (n = 130)	Total (n = 230)	NUD (n = 29)	IBS (n = 95)	Other (n = 106)	
% of patients with subjective improvement	55.2%	50%	44.8%	60.8%	48.3%	58.6%	43.2%	50.0%	
GSRS	33.6	34.8	34.4	32.5	31.8	31.5	33.0	31.0	
									Norm scores*
SCL-anxiety	18.4	15.8	19.4	18.0	16.9	15.5	17.2	17.0	12
SCL-depression	26.6	22.7	28.1	26.2	25.9	23.7	26.4	26.2	20
									Norm scores**
NHP-physical mobility	11.7	10.7	12.1	11.6	10.0	9.6	10.7	9.5	4.1
NHP-pain	20.3	16.1	21.5	20.3	14.7	11.0	16.2	14.3	7.0
NHP-sleep	20.9	14.8	26.4	17.4	16.1	13.4	20.3	13.1	15.2
NHP-energy	32.8	23.8	33.4	34.6	26.6	20.6	30.8	28.8	17.0
NHP-social isolation	10.2	6.3	12.4	9.3	7.8	7.2	6.9	8.7	5.3
NHP-emotional reactions	19.3	12.5	21.0	19.6	13.6	8.7	16.2	15.5	13.1

* Mean norm scores in a healthy population

** Mean norm scores: females 45–50 years of age

Table 2 shows that patients with long duration of complaints and without comorbidity at baseline had more improvement of their complaints at the end of the follow-up period. Patients with comorbidity scored more unfavourable on all items after follow-up. Age and sex were not related to subjective global improvement of complaints or to pain intensity. Patients older than 45 years of age scored higher on both NHP scales sleep and pain than patients 45 and younger. Female patients scored higher on pain intensity, NHP-pain and SCL subscale depression than male patients. Patients who had undergone therapeutic modalities scored significantly higher on both NHP scales sleep and pain and on SCL subscale depression than patients who were not.

**Table 2:** Relationship between baseline characteristics and outcome follow-up after 18 months (patient questionnaire data, N = 291)

*Groups*	*Improvement^a^*	*Pain intensity*		*Sleep (NHP)*		*Pain (NHP)*		*Anxiety (SCL)*		*Depression (SCL)*		*GSRS*	
	%	Score at follow-up	18 months change score^d^	Score at follow-up	18 months change score^d^	Score at follow-up	18 months change score^d^	Score at follow-up	18 months change score^d^	Score at follow-up	18 months change score^d^	Score at follow-up	18 months change score^d^
Age													
18 to 45	47.9	3.1	-0.6**	10.8	-3.4	9.7	-5.3	16.9	-0.9	25.4	-1.1**	31.1	-3.7**
45 to 70	48.5	3.5	-0.3	19.8	-2.3	18.2	-2.0	16.9	-0.5	26.4	+ 1.2	32.3	+ 0.5
Sex													
Male	45.8	2.8	-0.2	12.4	-3.1	10.8	-1.0	15.4	-0.4	23.4	0.0	31.0	-0.6
Female	49.7	3.6	-0.6	18.3	-2.6	16.5	-4.7	17.7	-0.9	27.4	+ 0.4	32.3	-1.6
Duration													
< 5 yrs.	54.5	3.2	-0.5	12.3	-3.3	12.8	-3.4	16.7	-0.9	25.5	+ 0.3	30.8	-2.0
> 5 yrs.	39.6*	3.5	-0.3	20.0	-1.6	15.9	-3.3	16.9	-0.5	25.9	0.0	32.5	+ 0.1
Comorbidity^c^													
Yes	39.8	3.9	-0.3**	21.1	-4.4	19.4	-1.5	18.4	-0.5	29.0	-0.2	34.0	-1.1
No	55.7	2.8	-0.6	11.7	-0.9	10.6	-5.6	15.6	-0.9	23.2	+ 0.8	29.9	-1.4
Diagnostics													
Yes	51.2	3.4	-0.9	17.8	-3.3	16.6	-5.1	16.8	-1.1	26.3	-0.4	32.1	-2.6
No	35.2	3.3	0.0	14.0	-2.0	12.1	-1.2	17.0	-0.2	25.5	+ 1.2	31.5	+ 0.6
Interventions													
Yes	50.0	3.5	-0.8	19.2	-2.2**	17.2	-5.2	17.3	-0.6	27.1	+ 0.3	32.5	-2.4
No	45.2	3.1	+ 0.3	10.7	-3.8	10.1	-0.1	16.2	-0.8	24.0	+ 0.3	30.6	+ 0.8
Referral													
Yes	36.8	4.3	0.0	16.4	+ 0.5	18.8	-7.3	17.6	-1.2	26.3	-1.6	36.7	+ 0.1
No	50.5	3.2	-0.3	16.1	-3.4	13.8	-2.6	16.8	-0.6	25.9	+ 0.7	30.9	-1.5

a. Percentage of patients that reported any improvement of their complaints at follow-up.

b. Pain intensity at follow-up as reported by the patients on an 11-point ordinal scale.

c. Comorbidity: patients who were suffering from a chronic disease other than non-specific abdominal complaints in the year before baseline and were treated for this chronic disease by their GP.

d. Absolute change scores; change between scores at baseline and follow-up

* p < 0.05, ** p < 0.01, both analyses based on the proportional change-score, in order to correct for differences in scores at baseline

Furthermore, patients who were referred scored higher on the GSRS than patients who were not referred.

When taking into consideration the proportional change scores (not in table), younger patients scored significantly lower on pain intensity, SCL subscale depression and GSRS after follow-up than patients older than 45 years. Based on the proportional change scores, patients without comorbidity scored significantly lower on pain intensity after follow-up than patients with comorbidity and patients who underwent therapeutic modalities scored significantly lower on NHP scale sleep than patients who did not.

Multivariable regression analysis however did not show any significant predictors of the course of chronic non-specific abdominal complaints.

### • Management of chronic non-specific abdominal complaints by family physicians

The GPs provided information on diagnosis, therapy and referrals during the 18 months follow-up and over the year previous to baseline. During the follow-up 50 per cent of all patients visited their GP because of chronic non-specific abdominal complaints, with a median number of visits of 1 (range 0–14). Physical examination was performed in 35.7% of the patients (n = 571), laboratory tests were done in 1.9%, X-rays were taken in 3.2% and endoscopy was used for 4.4% of the patients (Table 3). Whereas at baseline patients with NUD underwent significantly more endoscopies than patients with IBS or other abdominal complaints, after 18 months of follow-up no significant differences could be found. The largest proportion of patients that received medication were patients with NUD, although prescription of drugs in NUD-patients decreases from 66.7% at baseline to 40.0% after 18 months follow-up. The use of antacids in NUD decreases from 31.2% at baseline to 10.6% after 18 months, whereas the use of proton pump inhibitors doubles during follow-up (6.5% at baseline vs 12.9% after 18 months). Also the use of H2-receptor antagonists in NUD patients increases during follow-up (10.8% at baseline vs. 17.6% after 18 months). A similar pattern is seen in both other subgroups; the prescription of proton pump inhibitors and H2-receptor antagonists doubled after 18 months follow-up, although the use of both types of medication is significantly higher for NUD patients than for IBS patients. In the total population antispasmodics, proton pump inhibitors and H2-receptor antagonists were the most frequently used medication (16.6%, 11.0% and 12.1% respectively after 18 months). Only 9.3% of the patients were referred to secondary care by the GP during the 18 months follow-up period. The gastroenterologist is the specialist to whom most patients were referred.

**Table 3:** Diagnostic and therapeutic modalities in patients with chronic non-specific abdominal pain in general practice (reported by the GPs).^1^

	Baseline				1 1/2 years			
	Total (n = 619)	NUD (n = 93)	IBS (n = 244)	Other (n = 282)	Total (n = 571)	NUD (n = 85)	IBS (n = 230)	Other (n = 256)
Physical examination	42.0	47.3	37.7	44.0	35.7	41.2	36.5	33.2
Laboratory examination	11.6	7.5	9.4	15.0	1.9	0.0	2.6	2.0
X-ray	5.0	3.4	4.5	5.7	3.2	1.2	2.2	4.7
Endoscopy	6.3	15.1	3.3^a^	6.1^a^	4.4	3.5	4.3	4.7
								
Reassurance	25.4	15.1	26.6^a^	27.5^a^	22.4	18.8	24.8	21.5
Counseling concerning psychosocial stress	9.7	7.5	10.7	9.6	8.1	9.4	8.3	7.4
Dietary advice	14.5	14.0	18.0^c^	11.4	10.3	5.9	14.3	8.2
Medication	50.1	66.7	44.3	49.3	35.7	40.0	34.3	35.5
								
Paracetamol/aspirin	4.8	5.4	4.5	5.0	4.6	4.7	5.2	3.9
Antacids	16.0	31.2^b^	7.0^c^	18.2^a^	9.8	10.6	7.8	11.3
Antispasmodics	18.6	14.0	23.8^c^	15.7	16.6	9.4^b^	22.2^c^	14.1
Laxantia	5.0	5.4	4.1	5.0	3.3	1.2	4.3	3.1
Fiber supplements	5.5	1.1^b^	8.6^c^	3.9	5.8	1.2^b^	7.4	5.9
Antidepressants	3.7	3.2	3.7	3.9	2.6	3.5	3.0	2.0
Proton pump inhibitors	3.9	6.5	2.0	4.6	11.0	12.9^b^	5.2^c^	15.6
H2-receptor antagonist	6.9	10.8	4.1^a^	7.9	12.1	17.6^b^	8.7	13.3
Anti emetics	2.6	3.2	1.6	3.2	3.0	5.9	2.6	2.3

Percentage of patients who underwent at least once a specific diagnostic modality or were treated at least once with one therapeutic modality during the twelve-month period before baseline and during the 18 months prospective follow-up.

Note that since more than one modality can be applied in one patient, the sum of the column percentages may exceed 100%.

^a ^Significant difference with regards to NUD (p < 0.05)

^b ^Significant difference with regards to IBS (p < 0.05)

^c ^Significant difference with regards to Other (p < 0.05)

## Discussion

The aim of this study was to provide data on management and course of complaints of chronic non-specific abdominal pain in primary care. In this study, it was shown that after 18 months of follow-up 48.3% of the patients stated that their complaints have improved: in the NUD group this was 58.6% while in the IBS group this was 43.2%. In a previous review on the clinical course of NUD [9] we reported that for studies with a follow-up of 2 years or less the mean symptomatic improvement was 42.7% in NUD patients. In a review on the clinical course of IBS the improvement rate for IBS patients in studies with a follow-up of 3 years or less was 57% [8]. However, none of these studies has been done in general practice. This implies that in general, a substantial proportion of patients with chronic complaints about NUD and IBS in general practice show persistence of the symptoms over time. However it should be pointed out here that our results are applicable only to patients with already chronic disorders, and are invalid for all patients with IBS, NUD of FGID.

In our study, all patients showed anxiety and depression scores above norm scores, both at baseline and after follow-up. Patients with NUD had the lowest levels of anxiety and depression symptoms compared to patients with IBS and patients with other abdominal complaints.

The general health status of patients with chronic non-specific abdominal complaints was impaired at baseline and stayed almost unchanged after follow-up. Patients with IBS had a more impaired quality of life on all dimensions of the NHP than the other patients, and continued to have more impaired quality of life at the end of follow-up.

Information on the clinical course of chronic non-specific abdominal complaints was derived from the patient questionnaires, which were returned by 47% of the patients (after sending two reminders). This may imply that there is selection bias. Therefore we compared baseline characteristics and found differences on some potentially relevant patient characteristics between responding and non-responding patients. Responders had significantly shorter duration of complaints at the beginning of the study and visited the GP significantly more often than non-responders for abdominal complaints as well as in general. This may suggest that the responding patients comprise a subgroup of patients who more often actually suffered from their abdominal complaints at the moment they were asked to participate in this study. Non-responders possibly would have responded differently on questions on characteristics of complaints, general health perception and psychological assessment. We did not perform a non-response analysis at 18 months, but a similar bias may be present here.

Therefore, results regarding the clinical course of the complaints should be interpreted with some reservation. Nevertheless, because the entire study population, responders as well as non-responders, was used in describing the management of chronic non-specific abdominal complaints, the non-response did not have consequences for our estimates of the frequencies of the use of diagnostic modalities, therapeutic interventions and referrals.

After 18 months of follow-up we found that diagnostic investigations were not often used much. The diagnostic modality most often used was physical examination, whereas more expensive investigations like laboratory, X-ray, and endoscopic examination were used less frequently. Diagnostic modalities were most frequently applied in the group of patients with abdominal complaints, other than NUD and IBS. This may suggest that after the diagnostic procedure, there is less diagnostic uncertainty by the GPs during the follow-up of patients with NUD and IBS. Reassurance was still a frequently used therapy after 18 months of follow-up, especially in patients with IBS. This is in accordance with the current clinical guidelines for the management of IBS [22]. After 18 months of follow-up, medication was still prescribed in 35.7% of the patients. In patients with NUD the use of antacids was decreased whereas the use of proton pump inhibitors was increased. When taking into consideration that in IBS patients no single drug has been shown to be effective [23], a high proportion of patients still uses medication after 18 months follow-up.

In the follow-up period only 9.3% of the patients were referred to secondary or tertiary care. In case of referral, the gastroenterologist was the specialist most frequently referred to.

Our study population consisted of prevalent cases of chronic non-specific abdominal complaints. The patients had a mean duration of complaints of 5.2 years (range 1 to 41 years) at baseline. During follow-up no spectacular changes in the course of the complaints took place. In the bivariate analysis age, duration of complaints, comorbidity and appliance of therapeutic modalities seemed to be possible prognostic determinants. Since one of the aims of this study was to identify prognostic determinants of the course of the complaints, we planned to use a logistic regression model to investigate the association between the outcome measures improvement, NHP-subscales, SCL-subscales and GSRS and the various possible prognostic determinants. However, the data for the total cohort and several subgroups only showed small differences after follow-up, and multivariable logistic regression analysis did not show any significant prognostic determinants of the course of chronic non-specific abdominal complaints.

## Conclusion

This study shows that chronic non-specific abdominal complaints have a serious impact on the patients. The clinical course seems to be rather unfavourable, general health perception is clearly impaired and patients show high scores of anxiety and depression.

Since only few patients were referred to secondary or tertiary care, the GPs in our study seem to handle most of the patients with chronic non-specific abdominal complaints.

Relatively few diagnostic investigations were carried out. This suggests that once the diagnosis is determined, no indications for further investigations are found.

GPs prescribed relatively many drugs. There is a clear need for further evaluation of guidelines for management of chronic non-specific abdominal complaints and studies to investigate the implementation of these guidelines.

## Abbreviations

NUD: Non Ulcer Dyspepsia; IBS: Irritable Bowel Syndrome. FGID: Functional Gastro Intestinal Disease; GP: General Practitioner; SCL-90: Symptom Checklist 90; NHP: Nottingham Health Profile; GSRS: Gastrointestinal Symptom Rating Scale.

## Competing interests

The author(s) declare that they have no competing interests.

## Authors' contributions

all authors 1) have made substantial contributions to conception and design, or acquisition of data, or analysis and interpretation of data; 2) have been involved in drafting the manuscript or revising it critically for important intellectual content; and 3) have given final approval of the version to be published.

**Table 4:** Number of new referrals to medical specialists and allied care in patients with chronic non-specific abdominal complaints, as reported by the GPs.^1^

	Baseline				18 months			
	Total (n = 619)	NUD (n = 93)	IBS (n = 244)	Other (n = 282)	Total (n = 571)	NUD (n = 85)	IBS (n = 230)	Other (n = 256)
No referral	88.8	89.2	91.0	86.6	90.7	92.9	90.4	90.2
Gastroenterologist	6.6	6.5	6.1	7.1	5.3	2.4	5.7	5.9
Social worker	0.6	0.0	0.0	1.4	0.6	1.2	0.4	0.4
Dietician	0.5	1.1	0.0	0.7	0.9	0.0	0.9	1.2
Psychologist	1.1	2.2	1.6	0.4	0.7	0.0	0.9	0.8
Gynaecologist	1.6	0.0	1.2	2.5	0.5	0.0	0.4	0.8
Surgeon	0.5	1.1	0.4	0.4	0.9	1.2	1.7	0.0
Other	1.1	1.1	0.8	1.2	1.1	2.4	0.4	1.2

Percentage of patients who were referred at least once to a specific specialist during the twelve-month period before baseline and during the 1 1/2 years prospective follow-up. Note that since more than one modality can be applied in one patient, the sum of the column percentages may exceed 100%

## Pre-publication history

The pre-publication history for this paper can be accessed here:


